# Connect and Conquer: Collectivized Behavior of Mitochondria and Bacteria

**DOI:** 10.3389/fphys.2019.00340

**Published:** 2019-03-29

**Authors:** Catrin F. Williams, Christopher H. George

**Affiliations:** ^1^School of Engineering, Cardiff University, Cardiff, United Kingdom; ^2^Swansea University Medical School, Swansea, United Kingdom

**Keywords:** self-organization, mitochondria, collectivization, bacteria, dynamic system

## Abstract

The connectedness of signaling components in network structures is a universal feature of biologic information processing. Such organization enables the transduction of complex input stimuli into coherent outputs and is essential in modulating activities as diverse as the cooperation of bacteria within populations and the dynamic organization of mitochondria within cells. Here, we highlight some common principles that underpin collectivization in bacteria and mitochondrial populations and the advantages conferred by such behavior. We discuss the concept that bacteria and mitochondria act as signal transducers of their localized metabolic environments to bring about energy-dependent clustering to modulate higher-order function across multiple scales.

## Introduction

The conventional view of mitochondria as enslaved “powerhouses” of the cell has shifted to a more nuanced understanding of mitochondria as excitable, communicative entities that are enmeshed in complex signaling pathways to modulate an array of cellular processes (Whelan and Zuckerbraun, 2013; Chandel, 2015). Dynamic mitochondrial clustering and fragmentation (fusion and fission, respectively) influences events across multiple scales, for example, in cells (Ding et al., 2010), tissues (Familtseva et al., 2014) and organs (Eisner et al., 2017; Coronado et al., 2018) through to influencing the viability of the physiologic state (Biala et al., 2015) and even speciation (Barreto et al., 2018). These advances have helped evolve the evocative concept of mitochondria as pseudo-autonomous entities adopting “hive-like” order and behavior (Braschi and McBride, 2010).

There are striking similarities between the self-organization of mitochondrial networks and the collectivization of their ancestors, bacteria, into multicellular populations (e.g., biofilms). Both involve the transduction of localized environmental cues into altered function via the establishment and maintenance of extensively coupled networks (mitochondria) or matrix-embedded bacterial communities (biofilms). In the case of bacteria, selective pressures leading to the formation of biofilms result in emergent properties including resistance to environmental stressors and enhanced nutrient acquisition. These advantageous properties, which ultimately might directly influence higher-order behavior [e.g., between bacteria and their hosts (Hughes and Sperandio, 2008; Carding et al., 2015)], outweigh the high energetic costs of biofilm formation (Lyons and Kolter, 2015) (see section “The Benefits of the Impermanent Interactions”). Collectivization in bacterial biofilms thus represents exemplary self-organization and circular causality, i.e., bacteria generate their own local microenvironments (e.g., nutrient and oxygen gradients) that elicits responses on different temporal scales (e.g., by differential gene expression) which subsequently modulates localized conditions thereby tuning their ensuing behavior within the population etc., (Klauck et al., 2018; Piras et al., 2018).

Here, we consider evidence that mitochondria retain these hallmark features of topology, self-organization and feedback loops characteristic of bacterial collectivization. We highlight evidence that mitochondria, like bacteria, sense localized metabolic environments to bring about dynamic energy-dependent clustering events that entrain long-range correlations that subsequently modulate higher-order function across multiple scales.

## Self-Organization and Criticality

Biological networks are canonically “scale-free” and are defined by the presence of long-range, power-law correlations arising from multi-fractal connectedness operating over multiple spatial and temporal scales (Strogatz, 2000; Goldberger et al., 2002; Barabasi and Oltvai, 2004; Aon et al., 2008). Such network structures possess, at their core, autonomic self-organizing, self-repairing, and self-maintaining behavior. Thus the normal physiologic “steady state” is not one of constancy (as might be inferred by the term “homeostasis”), but rather is the manifestation of a dynamically configured system characterized by

(1) Multiple levels of control via delocalized “diffuse” coupled feedback loops,
(2) Spatio-temporal compartmentalization,
(3) Plasticity, reconfiguration/ adaptation,
(4) Intrinsic “memory” of previous configurations, and
(5) Energetic and entropic positioning far from equilibrium.

These properties are comprehensively reviewed elsewhere (Glass and Mackey, 1979; Ivanov et al., 1999; Goldberger et al., 2002; Weiss et al., 2006; Aon and Cortassa, 2012; Gintant and George, 2018).

The description of living cellular systems have been further refined to “dynamical entities that evolve and adapt with time and prior states having an influence on present states” (Bernabo et al., 2014). Here, we consider experimental evidence – which invokes the concept of “fractal dynamics” (Goldberger et al., 2002; Aon et al., 2008; Kurakin, 2011) – that such a description of higher-order network configuration would apply equally to bacteria and mitochondria.

**FIGURE 1:**
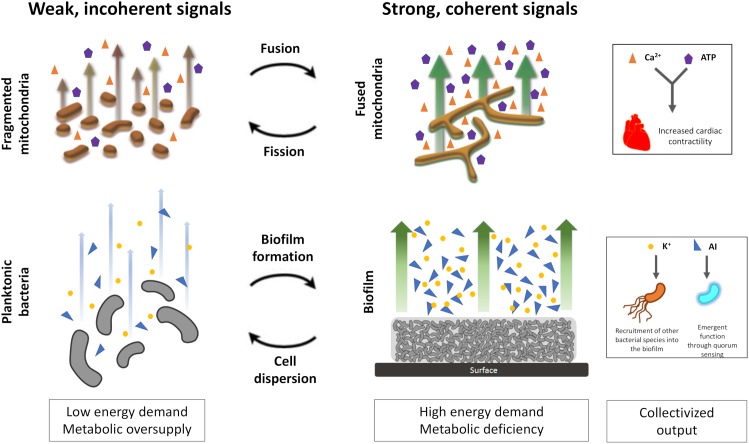
Collectivization and coherence in mitochondrial networks and bacterial biofilms. Under periods of low energy demand and nutrient sufficiency, mitochondria and bacteria tend to exist as individual units producing weak, incoherent signals. However, during periods of higher energy demand and lower nutrient availability, formation of coupled networks produces strong and coherent signaling. AI, autoinducers include modified peptides, hydroxylated pentanediones (“AI2”), derivatised lactones, heterocyclic compounds (e.g., quinolones, indoles) and esterified palmitic acid. Other AIs have been phenomenologically described as diffusible signal factor (DSF) and cholerae autoinducer-1 (CAI-1). The upper panel (mitochondria) is adapted from Picard and Burelle (2012) with permission.

The finely balanced physiologic network state enables rapid changes in functionality (e.g., increase in heart rate via the fight-or-flight mechanism) and longer-term adaptive responses (e.g., physiologic cardiac hypertrophy). Given the fractal nature of biological control, these same features of network topology also define lower-order behavior in bacteria and mitochondria (e.g., collectivization and altered behavior in response to changing nutrient availability and energy demands) (Figure 1).

In bacteria, nutrient availability promotes growth and biofilm establishment that is sustained by cooperativity and self-organization through propagation of electrical and metabolic signals (Liu et al., 2015; Lyons and Kolter, 2015; Prindle et al., 2015; Humphries et al., 2017). Feedback loops linking K^+^ channel opening and glutamate diffusion lead to periodic oscillatory growth that initiates suddenly when biofilms reach a critical size (i.e., existence close to points of bifurcation) (Liu et al., 2015; Prindle et al., 2015; Larkin et al., 2018). Similarly, criticality and percolation are also hallmark characteristics of mitochondria which have been shown to operate at the edge of dynamic instability (i.e., close to chaotic behavior) and undergo sharp phase transitions (Aon et al., 2004; Kurz et al., 2015; Zamponi et al., 2018).

## The Benefits of the Impermanent Interactions

Given the advantages conferred by physical interconnectivity as described above, it is pertinent to question why individualism has been retained in mitochondria and bacteria. While pseudo-reticular inter-mitochondrial connections do exist in skeletal muscle in order to facilitate energy distribution (Glancy et al., 2015), we consider some reasons why mitochondrial and bacterial interactions are dynamic and why this confers advantage over permanent fusion.

(i) Unlike permanent reticular structures in eukaryotes [e.g., endoplasmic reticulum (ER)], the spatiotemporal patterning of inter-mitochondrial interactions give rise to emergent properties in response to localized environments (e.g., redox and energetic status). Thus long-range ordering may be shaped by periodic oscillatory events that occur over numerous fission/fusion cycles and which influence behavior across multiple scales (see section “Introduction”). Similarly, it has been reported that oscillatory behavior associated with bacterial incorporation into biofilms encodes an adaptive “memory” that persists across multiple generations (Lee et al., 2018).
(ii) Collectivization is an energy-dependent process. Separateness enables the discrimination of individual units based on their functional competency (energetic “fitness”). Those mitochondria that are energetically damaged (i.e., “unfit” mitochondria with aberrant membrane potential), and thus do not have the requisite competency for fusion, are targeted for degradation via mitophagy (Twig et al., 2008; Vasquez-Trincado et al., 2016). Surveillance may thus serve as an early self-preservation mechanism to negate aberrant oscillatory behavior arising from the incorporation of damaged mitochondria into a mitochondrial network (Yang et al., 2015; Dey et al., 2018). This phenomenon has been suggested to modulate the longer-term viability of the host cell (Gomes et al., 2011). Likewise, surveillance of energetic fitness also exists to monitor the competency of individual bacteria to aggregate into multicellular structures (Burmann et al., 2011). Such quality control mechanisms ensure the fidelity of population behavior and enables the identification and elimination of “rogues” (Diggle et al., 2007; Allocati et al., 2015).
(iii) Although the modulation of mitochondrial networks is typically considered the product of fusion and fission events, mitochondrial nanotunnels have been discovered in heart cells where the densely packed cellular ultrastructure does not allow dynamic mitochondrial movement (Huang et al., 2013; Vincent et al., 2017). These nanotunnels support highly interconnected, non-fused networks of spatially segregated mitochondria. The evidence that inter-organellar tubular connections are conserved through evolution (Vincent et al., 2017), and also that heterogeneous mitochondrial distribution and function is a feature of non-excitable cells (Collins et al., 2002), suggests that nanotunnels may represent a common means to enhance mitochondrial network ordering in addition to fusion/fission events. In an intriguing corollary to the concept described in (ii) that the formation of mitochondrial networks represents the “selection of the fittest,” nanotunnels may form between those mitochondria exhibiting reduced competency for fusion or those showing early signs of damage (Vincent et al., 2017). Indeed, the observation of tubular protrusions may point to abortive fusion events by those energetically compromised mitochondria that are “reaching out for help” (Vincent et al., 2017). In a similar way, bacterial nanotubes enable sharing of nutrients and useful genes between distant neighbors, promoting cell survival (Dubey and Ben-Yehuda, 2011).

## On Quorum Sensing

The multi-step, energy-demanding process of biofilm formation is controlled by quorum sensing (QS). In this process, under conditions of nutrient availability, microbes sense their neighbors via autoinducers (AI) which results in the synchronization of gene expression across a microbial community (Nealson et al., 1970; Li and Tian, 2012; Figure 1). QS ensures bacterial collectivization when the propensity for emergent behavior is maximal, e.g., induction of bioluminescence (Nealson et al., 1970), enhanced virulence (Hentzer et al., 2003) and antibiotic production (Folcher et al., 2001). Opining on whether mitochondria exhibit QS, as has been posited by others (Picard and Burelle, 2012), is not the primary purpose of this Perspective. However, given the evidence above on the commonality of collectivization in bacteria and mitochondria, it is difficult to refute the existence of QS-like behavior in mitochondria. Notably, QS-like behavior is also observed in higher-order systems [e.g., the enhanced sensitivity to morphogenetic gradients in coupled populations of mammalian epithelial cells (Ellison et al., 2016)] and, remarkably, in artificial systems designed to produce autonomous devices (Hennig et al., 2015).

## Conclusion

It is an over-simplification to describe mitochondrial function as merely a response to environmental conditions and that mitochondrial “fission versus fusion” events are the final downstream acts of cellular commitment (“flourish” versus “die”). Rather, compelling evidence exists that the intrinsic behavior of mitochondria, via long-range correlation and chaotic behavior, contributes to setting the initial conditions to which they subsequently respond. By drawing parallels with the collectivized behavior of mitochondrial ancestors - bacteria - we conclude that dynamic remodeling of inter-mitochondrial interaction is a fundamental determinant of cellular fate andtissue function and that it critically influences higher-level ordering that underpins the physiologic state.

## Data Availability

No datasets were generated or analyzed for this study.

## Author Contributions

CW and CG jointly conceived, designed, and wrote the perspective, contributed to the manuscript revision, and read and approved the submitted version of the manuscript.

## Conflict of Interest Statement

The authors declare that the research was conducted in the absence of any commercial or financial relationships that could be construed as a potential conflict of interest.
